# Evaluation of the Easy Albumin–Bilirubin Score as a Prognostic Tool for Mortality in Adult Trauma Patients in the Intensive Care Unit: A Retrospective Study

**DOI:** 10.3390/diagnostics13223450

**Published:** 2023-11-15

**Authors:** Pao-Jen Kuo, Cheng-Shyuan Rau, Ching-Hua Tsai, Sheng-En Chou, Wei-Ti Su, Shiun-Yuan Hsu, Ching-Hua Hsieh

**Affiliations:** 1 Department of Plastic Surgery, Kaohsiung Chang Gung Memorial Hospital and Chang Gung University College of Medicine, Kaohsiung 83301, Taiwan; bow110470@gmail.com; 2 Department of Neurosurgery, Kaohsiung Chang Gung Memorial Hospital and Chang Gung University College of Medicine, Kaohsiung 83301, Taiwan; ersh2127@adm.cgmh.org.tw; 3 Department of Trauma Surgery, Kaohsiung Chang Gung Memorial Hospital and Chang Gung University College of Medicine, Kaohsiung 83301, Taiwan; tsai1737@cloud.cgmh.org.tw (C.-H.T.); athenechou@gmail.com (S.-E.C.); s101132@adm.cgmh.org.tw (W.-T.S.); ah.lucy@hotmail.com (S.-Y.H.)

**Keywords:** albumin–bilirubin, easy albumin–bilirubin, liver function, mortality, trauma, intensive care unit

## Abstract

The easy albumin–bilirubin (EZ–ALBI) score is derived using the following equation: total bilirubin (mg/dL) − 9 × albumin (g/dL). This study aimed to determine whether the EZ–ALBI score predicted mortality risk in adult trauma patients in an intensive care unit (ICU). Data from a hospital’s trauma database were retrospectively evaluated for 1083 adult trauma ICU patients (139 deaths and 944 survivors) between 1 January 2016 and 31 December 2021. Patients were classified based on the ideal EZ–ALBI cut-off of −26.5, which was determined via receiver operating characteristic curve analysis. The deceased patients’ EZ–ALBI scores were higher than those of the surviving patients (−26.8 ± 6.5 vs. −30.3 ± 5.9, *p* = 0.001). Multivariate logistic analysis revealed that, in addition to age, the presence of end-stage renal disease, Glasgow Coma Scale scores, and injury severity scores, the EZ–ALBI score is an independent risk factor for mortality (odds ratio (OR), 1.10; 95% confidence interval (CI): 1.06–1.14; *p* = 0.001)). Compared with patients with EZ–ALBI scores < −26.5, those with scores ≥ −26.5 had a 2.1-fold higher adjusted mortality rate (adjusted OR, 2.14; 95% CI: 1.43–3.19, *p* = 0.001). In conclusion, the EZ–ALBI score is a substantial and independent predictor of mortality and can be screened to stratify mortality risk in adult trauma ICU patients.

## 1. Introduction

The liver plays a crucial role in various physiological processes, such as metabolism, detoxification, and protein synthesis, all of which contribute to the maintenance of an individual’s health. Abnormal liver function can indicate the presence of systemic inflammation and malfunction; these factors have been linked to coagulopathy and sepsis, thus increasing the risk of mortality [1]. Indocyanine green clearance has been used to evaluate liver function; however, it is both resource-intensive and costly [2,3]. Additionally, although certain blood tests measuring liver enzymes are frequently employed in clinical practice, they may indicate the extent of liver injury or impairment rather than liver function reserve [4]. The albumin–bilirubin (ALBI) score, initially introduced through an international collaboration, was designed to evaluate liver function in hepatocellular carcinoma (HCC) patients [5]. Unlike traditional assessment tools, such as the Child–Turcotte–Pugh (CTP) classification [6] or the model for the end-stage liver disease (MELD) score [7], the ALBI score relies solely on albumin and bilirubin levels, making its calculation simpler. Its effectiveness has been confirmed in numerous studies for predicting outcomes in patients with advanced or resectable HCC [3,8,9,10,11]. Furthermore, it is valuable for assessing those with advanced HCC who are undergoing systemic therapy [9,10,11,12]. Notably, the applicability of the ALBI score has extended beyond HCC [13,14,15,16,17], thereby demonstrating its prognostic value in gastric cancer [18], lung cancer [19,20,21], esophageal cancer [22], and medulloblastoma [23]. Additionally, it is strongly associated with mortality in non-hepatologic conditions, such as aortic dissection [24], heart failure [25,26], acute pancreatitis [27], and trauma [28].

The ALBI score is computed using the following formula: ALBI score = (log_10_ bilirubin [µmol/L] × 0.66) + (albumin [g/L] × −0.0852). The ALBI grade is a useful and accurate predictor of outcomes in patients with HCC [9,29,30,31,32,33]. This includes individuals who are in the early and intermediate phases of the disease and those undergoing systemic interventions or HCC treatment. When assessing liver function in patients with HCC, the ALBI grade demonstrated greater predictive accuracy than the CTP score [34,35,36]. Higher ALBI grades were associated with a 2.06-fold increased risk of poor overall survival in patients with cancer who underwent liver resection [37]. Not only can the ALBI grade explain the risk of mortality in patients with moderate- or early-stage liver diseases who are undergoing liver resection [3], but it may also be applicable to trauma patients with liver injuries and may act as a surrogate for liver function [28]. According to the findings of a multivariate logistic regression study [28], the ALBI score was related to a mortality risk that increased by 2.42-fold in patients who suffered liver damage from trauma.

However, the complexity of this calculation limits its practical application. To address this issue, Ho et al. (2021) recently introduced a simplified EZ–ALBI score [37]. It replaced the ALBI score and was derived from serum albumin and bilirubin levels using a multivariate Cox proportional hazards model; it was calculated as total bilirubin (mg/dL) − 9 × albumin (g/dL) [38]. Notably, the EZ–ALBI score demonstrated a strong linear correlation (correlation coefficient, 0.965; *p* < 0.001) with the ALBI score across the entire patient cohort and in various subgroups, particularly in HCC patients [37]. Because of this simplified computation, the EZ–ALBI score is now a more accessible tool for monitoring the liver function of patients with liver disease undergoing various treatments [37,39,40,41].

This study aimed to investigate whether the EZ–ALBI score independently contributes to a mortality risk assessment, helping to stratify mortality risk in adult patients in the intensive care unit (ICU). This was performed based on the hypothesis that this score is associated with mortality risk in trauma and critically ill patients. The primary outcome of this study was the overall mortality rate in the study population.

## 2. Materials and Methods

### 2.1. Study Population and Data Acquisition

**Figure 1:**
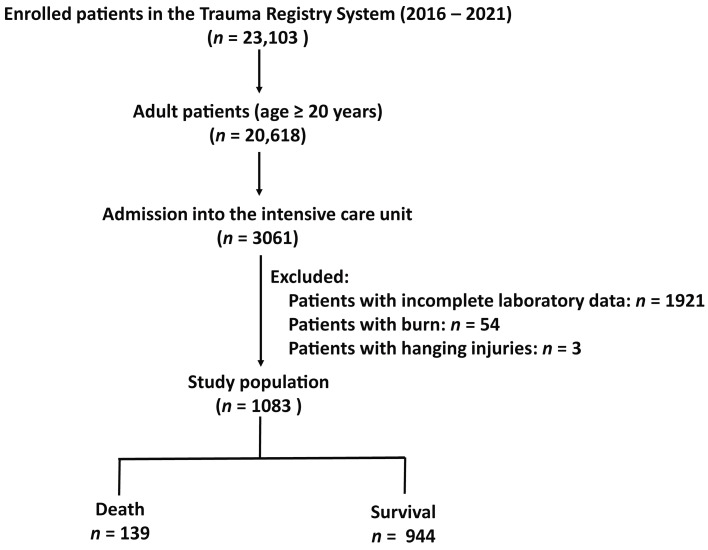
Flowchart depicting the process of selecting adult trauma patients with critical illnesses from the registered trauma database. Patients who were missing data on albumin or bilirubin levels or those with burn injuries or hanging injuries were excluded from the study group. The study population was subsequently divided into the following two groups: death and survival.

Between 1 January 2016 and 31 December 2021, there were a total of 23,103 hospitalized patients who sustained injuries from various causes of trauma and were admitted to the Trauma Registry System of Chang Gung Memorial Hospital [42] (Figure 1). Among these, there were 20,618 adult patients aged 20 years and older; 3061 patients were admitted to the ICU. Following the exclusion of patients with incomplete data regarding albumin or bilirubin levels (*n* = 1921) with burn injuries (*n* = 54), hanging injuries (*n* = 19), and those who drowned (*n* = 3), a study population of 1083 adult trauma patients was finally established. Data pertaining to this study group were extracted from a registered trauma database and included information such as sex, age, serum albumin and total bilirubin levels upon admission, pre-existing medical conditions, Glasgow Coma Scale (GCS) scores, injury severity scores (ISS), length of hospital stay, and in-hospital mortality. The easy albumin–bilirubin (EZ–ALBI) score was calculated using the following formula: total bilirubin (mg/dL) − 9 × albumin (g/dL).

### 2.2. Statistical Analyses

The two-sided Fisher’s exact test was used to evaluate categorical data. The Mann–Whitney U test was used for non-normally distributed continuous data; the findings were presented as medians with interquartile ranges (IQRs) between the first (Q1) and third (Q3) quartiles. When comparing continuous data with a normal distribution, an analysis of variance was used along with Bonferroni’s post hoc correction. The results were expressed as the mean ± standard deviation.

To study the determinants affecting patient mortality, univariate predictive variables were subjected to multivariate logistic regression analysis. The area under the curve (AUC) obtained from the receiver operating characteristic (ROC) curve was used to measure the efficacy of EZ–ALBI to predict patient death. The maximum Youden index (sensitivity + specificity − 1) obtained from the ROC curve was used to identify the appropriate cut-off value for EZ–ALBI.

Patients were separated into two groups according to the cut-off point. Their mortality risk was compared using adjusted odds ratios (AORs) and 95% confidence intervals (CIs) as determined via logistic regression analysis. All statistical analyses were performed using SPSS Statistics software (version 23.0; IBM; *p* = 0.05).

## 3. Results

### 3.1. Injury and Patient Characteristics between Death and Survival Patients

An analysis comparing the 139 deceased and 944 surviving patients revealed several distinctions (Table 1). First, the deceased patients were of an advanced age compared to those who survived. In addition, deceased patients exhibited a notably higher EZ–ALBI score (−26.8 ± 6.5) in contrast to those who survived (−30.3 ± 5.9, *p* < 0.001). Furthermore, the deceased patients also had a significantly lower serum albumin level (3.1 ± 0.8) compared to those who survived (3.5 ± 0.7, *p* < 0.001); however, there was no substantial difference in total bilirubin levels between these two groups.

Regarding pre-existing health conditions, there were considerably higher rates of comorbidities, such as hypertension (HTN), coronary artery disease (CAD), congestive heart disease (CHF), and end-stage renal disease (ESRD), in the deceased patients than in those who survived. Deceased patients also presented with notably lower Glasgow Coma Scale (GCS) scores (median [IQR, Q1–Q3], GCS:7 [3–14]) but higher injury severity scores (ISS) (25 [20–33]) than individuals who survived (GCS:15 [9–15], ISS:20 [16–25]; *p* < 0.001). The patients who died had a significantly shorter length of stay in the ICU than those who survived (5.2 days vs. 13.6 days; *p* < 0.001). Additionally, deceased patients experienced a considerably shorter hospitalization period (15.7 days) than individuals who survived (23.2 days; *p* < 0.001).

### 3.2. Analysis of the Risk Factors for Mortality

In the univariate analysis, various factors were found to be statistically significant risk factors for mortality in the study population. These factors included age, the EZ–ALBI score, HTN, CAD, CHF, ESRD, GCS score, and ISS (Table 2). In the multivariate logistic regression analysis, it was shown that EZ–ALBI (odds ratio [OR], 1.10; 95% CI, 1.06–1.14; *p* < 0.001) emerged as a statistically significant independent risk factor for mortality. Furthermore, our analysis revealed that age (OR, 1.03; 95% CI, 1.02–1.04; *p* < 0.001), ESRD (OR, 2.91; 95% CI, 1.26–6.71; *p* = 0.012), GCS (OR, 0.85; 95% CI, 0.81–0.89; *p* < 0.001), and ISS (OR, 1.07; 95% CI, 1.04–1.09; *p* < 0.001) were significant independent risk factors for mortality in this cohort. HTN and CHF were not significant independent risk factors for mortality in trauma patients in the ICU.

### 3.3. The Outcomes of Patients Divided into Two Categories Based on the EZ–ALBI Cut-Off Value

**Figure 2:**
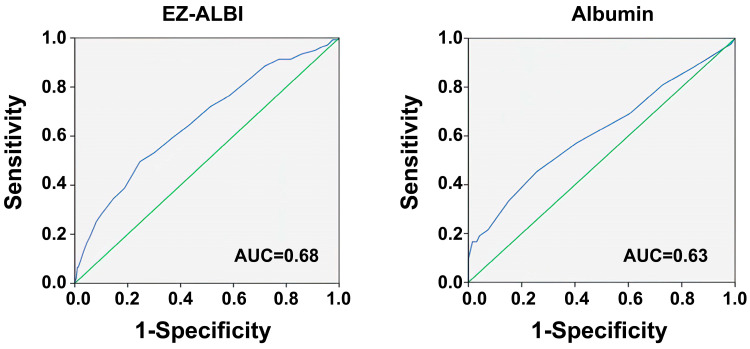
The area under the curve (AUC) and receiver operating characteristic curves (ROC) of the EZ–ALBI score and albumin alone for predicting the mortality of adult trauma patients in the intensive care unit.

Based on the ROC curve analysis, an EZ-ALBI score of −26.5 was determined as the optimal cut-off point, which demonstrated the highest AUC value of 0.68. At this cut-off, the sensitivity and specificity were 0.516 and 0.753, respectively (Figure 2). As indicated in Table 3, patients with an EZ–ALBI score ≥ −26.5 were primarily male and notably older compared to those with an EZ–ALBI score < −26.5. Among patients with an EZ-ALBI score ≥ −26.5, there was a significantly higher prevalence of pre-existing ESRD, although there were no notable differences in other comorbidities when compared to those with an EZ-ALBI score < −26.5. However, albumin alone had the highest AUC value of 0.63 when predicting the outcome, with a cut-off value of 2.95 g/dL. The ability of EZ–ALBI to predict patient mortality was moderately accurate and substantially superior to that of albumin alone (*p* = 0.048).

Furthermore, patients with an EZ–ALBI score ≥ −26.5 presented with a significantly lower GCS score (14 [6–15] vs. 15 [9–15], p = 0.001) and a higher ISS score (25 [16–29] vs. 20 [16–25], *p* < 0.001) than individuals with an EZ–ALBI score < −26.5. Importantly, patients with an EZ–ALBI score ≥ −26.5 exhibited a substantially higher mortality rate than individuals with an EZ–ALBI score < −26.5 (22.8% vs. 9.0%, *p* < 0.001). Even after adjusting for sex, age, pre-existing ESRD, GCS, and ISS, patients with an EZ–ALBI score ≥ −26.5 still showed a significantly higher adjusted mortality rate (AOR, 2.14; 95% CI: 1.43–3.19, *p* < 0.001) compared to patients with an EZ–ALBI score < −26.5.

## 4. Discussion

The ALBI score combines two essential markers of the liver’s metabolic (bilirubin) and synthetic (albumin) activities [43]. Patients who have suffered trauma, particularly from accidents, falls, or acts of violence, may experience a heightened loss of red blood cells, thereby resulting in increased bilirubin levels [44]. Hyperbilirubinemia in critically ill patients can be attributed to various factors, such as biliary obstruction, liver disease, hemolysis, hematoma resorption, and medication toxicity [44,45,46]. The liver is responsible for the primary processing and elimination of bilirubin. In instances of significant trauma, particularly those affecting the abdominal region or liver, there is the potential for compromised hepatic bilirubin metabolism. Elevated bilirubin levels are indicative of liver malfunction, which has the potential to exacerbate a patient’s overall disease status [45,46]. Elevated levels of bilirubin may serve as an indicator of tissue damage and the degree of injury [44]. Nevertheless, trauma patients may develop hyperbilirubinemia even in the absence of a pre-existing hepatobiliary illness. The primary etiology of jaundice in individuals with traumatic injuries is commonly linked to variables such as initial hypoperfusion, systemic hypotension, blood transfusion, and hematoma formation [47,48]. Elevated bilirubin levels in trauma patients serve as a potential indicator of unfavorable prognosis. Additionally, the presence of persistent or increasing lactate levels throughout a patient’s ICU course may indicate a higher probability of complications, organ failure, or fatality [45,46]. Hence, bilirubin has been employed as a metric to assess liver impairment in critically ill patients, thus functioning as an indicator of liver performance in various prognostic models, such as the Sequential Organ Failure Assessment (SOFA) score [49], the Acute Physiology and Chronic Health Evaluation (APACHE) score [50], the Simplified Acute Physiology Score (SAPS II) [51], the Multiple Organ Dysfunction Score (MODS) [52], and the Logistic Organ Dysfunction Score (LODS) [53]. According to previous estimates, hyperbilirubinemia was found to affect approximately 40% of critically ill patients and was linked to increased mortality rates and unfavorable outcomes [54,55]. In this study, although the total bilirubin levels of surviving patients did not differ significantly different from those of the deceased patients in this study, the sole ability of EZ–ALBI to predict patient mortality was moderately accurate and substantially superior to that of albumin alone (*p* = 0.048).

Conversely, albumin is a liver-produced protein that plays a role in preserving the intravascular volume by mediating oncotic pressure [56,57], which is crucial for maintaining a balance of fluids in the body. Hypoalbuminemia may indicate malnutrition or inflammation, which are conditions frequently observed in admitted patients [58]. Malnutrition can undermine the body’s capacity to recover from injury, whereas inflammation can accelerate albumin breakdown. Insufficient albumin levels may contribute to fluid imbalances, thus potentially causing edema, affecting cardiac function, and promoting factors associated with less favorable outcomes in trauma patients [56,57,59,60]. Additionally, albumin levels may reflect the severity of the injury and overall physiological stress, thereby serving as a predictive marker for complications, an extended ICU stay, and increased mortality risk [58]. Nevertheless, unlike bilirubin, albumin levels are not typically considered a primary variable in most ICU prediction models.

In the context of clinical practice, the evaluation of liver function and overall health often involves the measurement of albumin and bilirubin levels. Although they fulfill distinct purposes and signify various facets of well-being, there are instances that enable them to offer a more all-encompassing evaluation of an individual’s state. In contrast to alternative methodologies, the ALBI score mitigates inter-observer variability by exclusively utilizing objective laboratory measurements of albumin and bilirubin [61]. The equation used for calculating the EZ–ALBI score demonstrated a higher level of simplicity than that of the ALBI score alone while still exhibiting a strong correlation with the latter in predicting prognosis [38], post-procedural survival following transarterial chemoembolization [39] and radiofrequency ablation [40] in HCC patients. Furthermore, ALBI and EZ–ALBI grades have been identified as viable indicators of liver failure and are capable of categorizing the long-term survival rates of patients who have ascites [62]. The EZ–ALBI score could be a beneficial screening tool for identifying adult trauma patients with a high risk of mortality, owing to its ease of assessment.

In this study, individuals who died had significantly higher EZ–ALBI scores than individuals who survived. Individuals with EZ-ALBI scores ≥ −26.5 had a roughly 2.1-fold greater adjusted mortality rate compared to patients with EZ-ALBI scores < −26.5. These data highlight the importance of the EZ–ALBI score as an independent risk factor for mortality in adult trauma patients, regardless of the underlying cause. However, it should be noted that at optimal EZ–ALBI cut-offs, the sensitivity was 0.516, the specificity was 0.753, and the greatest AUC value was not particularly high (AUC = 0.68). Therefore, EZ–ALBI is only suitable for screening patients with a high risk of mortality but may be used in conjunction with other evaluation tools because of its simplicity. Consequently, the EZ–ALBI score could be a valuable tool for stratifying the mortality risk in trauma patients with critical illness.

The severity of liver dysfunction is often estimated using the CTP classification or the MELD score [6]. Based on the five variables of ascites, hepatic encephalopathy, albumin, prothrombin time, and total bilirubin, the CTP classification is a valuable tool for assessing liver disease severity [63]. The CTP classification includes subjective components, such as hepatic encephalopathy and ascites, which may lead to significant variations in the scoring among healthcare providers [64]. The CTP classification is primarily designed to assess cirrhosis, which limits its applicability to other liver diseases; there were over 30 variations in the CTP categorization, thus causing errors in scoring [65]. The MELD score, on the other hand, is a continuous value generated from the levels of serum creatinine, bilirubin, and the international normalized PT ratio [66,67,68]. The use of the MELD score in patients with milder liver impairment has drawn criticism [5]; it was primarily created for end-stage cirrhosis patients or those awaiting liver transplantation [69,70,71]. The MELD score relies heavily on serum creatinine levels, wherein small changes in kidney function can have a large impact on the overall value [72]. Furthermore, the precision and reliability of MELD scores are considerably affected by variations in international normalized ratios across different laboratories [72,73,74]. Additionally, it has been discovered that the ALBI score performed better than CTP in classifying different prognostic subgroups within CTP [43]. Thus, future studies could be conducted by investigating the relationship between the EZ–ALBI score and mortality risk in patients with various medical conditions, assessing the utility of the EZ–ALBI score to predict long-term mortality outcomes in trauma patients. This includes analyzing the dynamic changes in EZ–ALBI scores during hospitalization, comparing the predictive accuracy of the EZ–ALBI score with other classification systems, such as CTP or the MELD, exploring the possibility of incorporating additional laboratory or clinical variables into the EZ–ALBI score to enhance its predictive value, and conducting a multicenter study to validate the findings of the current study and evaluate the generalizability of the EZ–ALBI score as a screening tool for stratifying mortality.

This study demonstrates that the EZ–ALBI score could be a valuable tool for stratifying the mortality risk in trauma patients with critical illnesses. However, this study has certain limitations. First, we only examined hospital mortality rates, excluding fatalities that occurred after admission to the emergency room and patients with long-term mortality. Thus, when comparing the results, selection bias may have occurred. Furthermore, data were obtained prospectively as part of the mandated trauma registry process. However, the retrospective nature of this study presented the possibility of selection bias, as it may have excluded individuals who did not have complete total bilirubin and albumin data. The dynamic changes in bilirubin and albumin levels in trauma patients during hospitalization may provide useful information; however, it was not possible to record these changes during this study. Furthermore, the outcomes found in the study population may have been influenced by variations in interventions, such as the type and volume of the fluid or blood component replacement during resuscitation, damage control performance, and surgical procedures. Nonetheless, it was assumed that the effects of these therapies were consistent across the study cohort. Finally, the research analysis predominantly focused on a single urban trauma center, thereby limiting the extent to which these findings can be generalized to other geographical locations.

## 5. Conclusions

The findings of this study indicate that the EZ–ALBI score is a significant independent risk factor for mortality risk. Therefore, it can be considered a valuable screening tool for stratifying the mortality risk in adult trauma patients admitted to the ICU.

## Figures and Tables

**Table 1:** Comparison of the injuries and patient characteristics of death and survival patients in the study population.

Variables	Death *n* = 139	Survival *n* = 944	OR (95% CI)	*p*
Gender				0.143
Male, *n* (%)	101(72.7)	627(66.4)	1.34(0.90–2.00)	
Female, *n* (%)	38(27.3)	317(33.6)	0.74(0.50–1.11)	
Age, years (SD)	61.6 ± 19.3	55.3 ± 19.4		<0.001
EZ–ALBI	−26.8 ± 6.5	−30.3 ± 5.9		<0.001
Albumin (g/dL)	3.1 ± 0.8	3.5 ± 0.7		<0.001
Total bilirubin (mg/dL)	1.1 ± 1.1	1.2 ± 2.0		0.824
Comorbidities				
CVA, *n* (%)	4(2.9)	43(4.6)	0.62(0.22–1.76)	0.365
HTN, *n* (%)	58(41.7)	301(31.9)	1.53(1.06–2.20)	0.021
CAD, *n* (%)	19(13.7)	79(8.4)	1.73(1.01–2.96)	0.042
CHF, *n* (%)	4(2.9)	5(0.5)	5.56(1.48–20.98)	0.004
DM, *n* (%)	33(23.7)	199(21.1)	1.17(0.77–1.78)	0.475
ESRD, *n* (%)	12(8.6)	29(3.1)	2.98(1.48–5.99)	0.001
GCS, median (IQR)	7(3–14)	15(9–15)		<0.001
ISS, median (IQR)	25(20–33)	20(16–25)		<0.001
1–15	11(7.9)	222(23.5)	0.28(0.15–0.53)	<0.001
16–24	31(22.3)	403(42.7)	0.39(0.25–0.59)	<0.001
≥25	97(69.8)	319(33.8)	4.53(3.08–6.66)	<0.001
ICU stay, days (SD)	5.2 ± 6.1	13.6 ± 11.4		<0.001
Hospital LOS, days (SD)	15.7 ± 16.8	23.2 ± 17.8		<0.001

CAD, coronary artery disease; CHF, congestive heart failure; CI, confidence interval; CVA, cerebral vascular accident; DM, diabetes mellitus; EZ–ALBI, easy albumin–bilirubin; ESRD, end-stage renal disease; GCS, Glasgow Coma Scale; HTN, hypertension; IQR, interquartile range; ISS, injury severity score; LOS, length of stay; OR, odds ratio; SD, standard deviation.

**Table 2:** Univariate and multivariate analysis of the risk factors for mortality in ICU patients.

Variables	Univariate Analysis			Multivariable Analysis		
	OR	CI	*p*	OR	CI	*p*
Age, years	1.02	(1.01–1.03)	<0.001	1.03	(1.02–1.04)	<0.001
EZ–ALBI	1.10	(1.06–1.13)	<0.001	1.10	(1.06–1.14)	<0.001
HTN, yes	1.53	(1.06–2.20)	0.022	1.21	(0.75–1.95)	0.434
CAD, yes	1.73	(1.01–2.96)	0.044	1.75	(0.91–3.37)	0.093
CHF, yes	5.56	(1.48–20.98)	0.011	1.36	(0.30–6.26)	0.692
ESRD, yes	2.98	(1.48–5.99)	0.002	2.91	(1.26–6.71)	0.012
GCS	0.84	(0.81–0.87)	<0.001	0.85	(0.81–0.89)	<0.001
ISS	1.07	(1.05–1.09)	<0.001	1.07	(1.04–1.09)	<0.001

CAD, coronary artery disease; CHF, congestive heart failure; CI, confidence interval; EZ–ALBI, easy albumin–bilirubin; ESRD, end-stage renal disease; HTN, hypertension; GCS, Glasgow Coma Scale; ISS, injury severity score; OR, odds ratio.

**Table 3:** The outcomes of patients divided into two categories based on the EZ–ALBI cut-off value.

Variables	EZ–ALBI ≥ −26.5 *n* = 302	EZ–ALBI < −26.5 *n* = 781	OR (95%CI)	*p*
Gender				0.021
Male, *n* (%)	219(72.5)	509(65.2)	1.41(1.05–1.89)	
Female, *n* (%)	83(27.5)	272(34.8)	0.71(0.53–0.95)	
Age, years (SD)	59.0 ± 18.3	55.0 ± 19.8		0.002
Comorbidities				
CVA, *n* (%)	8(2.6)	39(5.0)	0.52(0.24–1.12)	0.089
HTN, *n* (%)	108(35.8)	251(32.1)	1.18(0.89–1.55)	0.256
CAD, *n* (%)	23(7.6)	75(9.6)	0.78(0.48–1.26)	0.307
CHF, *n* (%)	5(0.7)	4(0.5)	3.27(0.87–12.26)	0.063
DM, *n* (%)	63(20.9)	169(21.6)	0.96(0.69–1.32)	0.780
ESRD, *n* (%)	17(5.6)	24(3.1)	1.88(1.00–3.55)	0.048
GCS, median (IQR)	14(6–15)	15(9–15)		0.001
ISS, median (IQR)	25(16–29)	20(16–25)		<0.001
1–15	49(16.2)	184(23.6)	0.63(0.44–0.89)	0.008
16–24	99(32.8)	335(42.9)	0.65(0.49–0.86)	0.002
≥25	154(51.0)	262(33.5)	2.06(1.57–2.70)	<0.001
Mortality, *n* (%)	69(22.8)	70(9.0)	3.01(2.09–4.33)	<0.001
Mortality AOR			2.14(1.43–3.19)	<0.001

AOR, adjusted odds ratio; CAD, confidence interval; CHF, coronary artery disease; CI, confidence interval; CVA, cerebrovascular accident; DM, diabetes mellitus; ESRD, end-stage renal disease; GCS, Glasgow Coma Scale; HTN, hypertension; IQR, interquartile range; ISS, injury severity score; OR, odds ratio; SD, standard deviation. The AOR of mortality was calculated after adjusting for sex, age, preexisting ESRD, the GCS score, and ISS.

## Data Availability

Data are contained within the article.
